# Interscalene brachial plexus block for outpatient shoulder arthroplasty: Postoperative analgesia, patient satisfaction and complications

**DOI:** 10.4103/0019-5413.33688

**Published:** 2007

**Authors:** Anand Shah, Karen C Nielsen, Larissa Braga, Ricardo Pietrobon, Stephen M Klein, Susan M Steele

**Affiliations:** School of Medicine, University of Pennsylvania, Philadelphia, PA, USA; * The Center for Excellence in Surgical Outcomes, University of Nebraska Medical Center, Omaha, NE; # The Division of Ambulatory Anesthesia, Department of Anesthesiology, Duke University Medical Center, Durham, North Carolina, USA; § The Division of Orthopedic Surgery, Department of Surgery, Duke University Medical Center, Durham, North Carolina, USA

**Keywords:** Analgesia, interscalene brachial plexus block, regional anesthesia, satisfaction, shoulder arthroplasty

## Abstract

**Background:**

Shoulder arthroplasty procedures are seldom performed on an ambulatory basis. Our objective was to examine postoperative analgesia, nausea and vomiting, patient satisfaction and complications of ambulatory shoulder arthroplasty performed using interscalene brachial plexus block (ISB).

**Materials and Methods:**

We prospectively examined 82 consecutive patients undergoing total and hemi-shoulder arthroplasty under ISB. Eighty-nine per cent (n=73) of patients received a continuous ISB; 11% (n=9) received a single-injection ISB. The blocks were performed using a nerve stimulator technique. Thirty to 40 mL of 0.5% ropivacaine with 1:400,000 epinephrine was injected perineurally after appropriate muscle twitches were elicited at a current of less than 0.5% mA. Data were collected in the preoperative holding area, intraoperatively and postoperatively including the postanesthesia care unit (PACU), at 24h and at seven days.

**Results:**

Mean postoperative pain scores at rest were 0.8 ± 2.3 in PACU (with movement, 0.9 ± 2.5), 2.5 ± 3.1 at 24h and 2.8 ± 2.1 at seven days. Mean postoperative nausea and vomiting (PONV) scores were 0.2 ± 1.2 in the PACU and 0.4 ± 1.4 at 24h. Satisfaction scores were 4.8 ± 0.6 and 4.8 ± 0.7, respectively, at 24h and seven days. Minimal complications were noted postoperatively at 30 days.

**Conclusions:**

Regional anesthesia offers sufficient analgesia during the hospital stay for shoulder arthroplasty procedures while adhering to high patient comfort and satisfaction, with low complications.

Interscalene brachial plexus blockade (ISB) has been shown to be an effective anesthetic technique for inpatient shoulder surgery.^1^–^3^ Compared to patients receiving general anesthesia, these patients have shorter hospital stays^4^ and a reduced need for postoperative analgesics.^5^,^6^ Patients undergoing ISB also experience less time in the postanesthesia care unit (PACU)^6^–^8^ and importantly, high levels of satisfaction with their anesthesia.^7^,^9^–^11^ In studies of patients undergoing shoulder surgery procedures, continuous ISB with an infusion of local anesthetic has been shown to have the added advantage of prolonging the period of analgesia after surgery^3^,^12^–^14^ while decreasing postoperative opioid requirements.^13^

Nevertheless, there are a few descriptions of analgesia and patient satisfaction related to regional anesthesia used in the ambulatory (outpatient) setting for shoulder arthroplasty procedures. Ilfeld *et al.,* recently examined the feasibility of total shoulder arthroplasty (TSA) on an outpatient basis using a perineural local anesthetic infusion in 14 patients.^15^ Although the results were encouraging, the small sample size warranted further study with a larger population. We analyzed a series to prospectively examine postoperative analgesia, postoperative nausea and vomiting (PONV), patient satisfaction and complications of ISB in patients undergoing outpatient shoulder arthroplasty procedures.

## MATERIALS AND METHODS

This study was approved by our Institutional Review Board (IRB). All consecutive patients undergoing TSA or shoulder hemiarthroplasty (HSA) procedures at our institution's ambulatory surgery center (ASC) during a 24-month interval were included in this prospective study. One surgeon performed all surgical procedures and four anesthesiologists provided anesthesia. Demographic, anesthesia and surgical data was prospectively entered into an electronic ambulatory anesthesia database. Demographic data collected included age, gender, American Society of Anesthesiologists (ASA) physical status classification and co-morbid illnesses. Intraoperative data included surgical time, blood transfusion requirements and hemodynamic data. The regional anesthesia technique and use of local anesthetics were also documented. Blocks were classified as continuous ISB or single-injection ISB. In addition, patients were asked questions regarding their pain, PONV, satisfaction and self-reported complications, in the PACU and at fixed intervals in the postoperative period; patients were asked both to describe any complications or symptoms they may be experiencing and also to rate their pain, PONV and satisfaction according to the scales described below. Physicians and research nurses collected data in the PACU and at 24h postoperatively in person. In addition, research nurses collected postoperative data by telephone at seven days. If the patient was unavailable at the time of the initial phone call, a second call was placed one day later. Patients unavailable by phone contact received a written questionnaire by mail. In addition, we contacted the patient by telephone and assessed the medical record at 30 days for any complications resulting from the procedure, including those not related to anesthesia. Anesthetic complications that were assessed included, but were not limited to: residual paresthesias, residual motor deficits, pain, infection and swelling or bruising at the block injection site.

In the PACU, at 24h and seven days postoperatively, patients were asked to record their pain at the surgical site using a verbal analog pain score (VAPS 0=no pain/10=worst pain imaginable). The VAPS was assessed both at rest and with movement in the PACU. At 24h and seven days, patients were asked to rate their overall pain. Efficacy of the block (based on the need for postoperative opioids) as well as disposition (either home or scheduled inpatient stay; Table 1) from the PACU was also recorded. The PONV verbal analog scores (0 = no nausea / 10 = vomiting) were collected in the PACU and at 24h using a modified 10-point scale that has been previously described.^16^ In addition, overall patient satisfaction with anesthesia was also obtained at 24h and seven days using a satisfaction scale (1 = very dissatisfied / 5 = extremely satisfied) that has been previously used at our institution in several prospective studies.^17^,^18^ Satisfaction surveys were administered to all patients, including those with a planned combined approach and patients who were subsequently converted to general anesthesia.

**Table 1:** Demographic, anesthesia and surgical characteristics

	All patients	TSA (n=64)	HSA (n=18)	*P*
Age (years)	65.1 ± 12.4	63.9 ±12.7	69.5 ±10.1	NS
Gender (female, %)	62.2	56.2	83.3	0.04
Race (white, %)	93.4	91.7	100	NS
ASA class (%)				NS
II	51.3	58.3	27.8	
III	44.9	38.3	66.7	
IV	3.9	3.3	5.6	
BMI (kg/m^2^)	30.2 ±6.1	30.1±5.7	30.8 ± 7.6	NS
Primary technique (%)				NS
Combined regional/GA (planned)	11.0	10.9	11.1	
Regional only	89.0	89.1	88.9	
Type of block (%)				NS
Continuous interscalene block	89.0	90.6	83.3	
Single-injection interscalene block	11.0	9.4	16.7	
Quality of block (%)				NS
Failed (required GA)	8.5	9.4	5.6	
Inadequate (required local)	1.2	1.6	0.0	
Inadequate (required re-block)	6.1	7.8	0.0	
Planned combined	11.0	10.9	11.1	
Surgical	73.2	70.3	83.3	
Side of surgical procedure (%)				NS
Left	45.4	44.1	50.0	
Right	54.6	55.9	50.0	
Actual surgical time (min)	214 ±34	220 ± 31	192 ±34	<0.01
Disposition (%)				NS
Home	84.9	86.8	83.3	
Inpatient (scheduled)	15.1	13.1	16.7	
Postanesthesia care unit time (min)	133 ± 111	131 ± 16	139 ± 20	NS

TSA = Total shoulder arthroplasty; HSA = Shoulder hemiarthroplasty. GA = General anesthesia, Mean *±* standard deviation, min = Minutes

### Ambulatory anesthesia techniques

All patients received either: 1) continuous ISB or a single-injection ISB as a primary anesthetic or 2) planned combined regional (either single-injection ISB or continuous ISB) and general anesthesia. Anesthetic technique was determined at the discretion of the anesthesiologist. Most of the patients (89%) received a continuous ISB, as this is the standard of care for major open shoulder procedures at our outpatient surgery center. However, the attending anesthesiologist made the final decision according to patient history, physical examination and other perioperative considerations. All regional anesthetic techniques were performed in a preoperative holding area and monitored using standard American Society of Anesthesiologists (ASA) monitors for blood pressure (noninvasive), heart rate, pulse oximetry and capnography. Patients were sedated with intravenous midazolam (1-5 mg) and fentanyl (50-250 mcg), titrated to moderate sedation (arousable on command). These medications were used in the preoperative holding area for block placement. All blocks were performed using the approach previously described by Winnie.^19^ Single-injection ISB was performed using a 22-gauge, 50 mm insulated, blunt needle (B. Braun Medical, Bethlehem, PA) and a nerve stimulator; a stimulus was sought distal to the shoulder. After an appropriate stimulus was localized with a current, less than 0.5 mA, 30-40 ml of 0.5% ropivacaine (Naropin“, Astra Pharmaceuticals, Westborough, MA) with 1:400,000 epinephrine was injected in 3-5 mL increments. Continuous ISB were performed using the same technique via an 18-gauge, 3.81 cm insulated Tuohy needle (Contiplex, B. Braun Medical, Bethlehem, PA). After stimulating an appropriate muscular response and injecting the local anesthetic, the needle was maintained in the same position and a 20-gauge standard epidural catheter was threaded 4-5 cm into the sheath of the brachial plexus. Catheters were secured with medical adhesive, cutaneous adhesive sutures (Ethicon, Somerville, NJ) and an occlusive dressing. Patients undergoing continuous ISB received a perineural infusion of 0.2% ropivacaine at a rate of 10 mL/h during the 23h observation unit stay. In the morning of postoperative Day 1, prior to patient discharge, perineural catheters were redosed with 20 mL of 0.5% ropivacaine with epinephrine 1:400,000 and then removed.

Opioids were used again in the postoperative period (in the PACU and 23h observation unit) if patients had pain. As per the standard of practice at our outpatient surgery center, in patients with VAPS 3 to 5, oral opioids were used (oxycodone 5-10 mg PO). If patients had pain VAPS 6 to 10, IV opioids were used (morphine or fentanyl). Nurses in the 23h observation unit assess pain scores every two to four hours. [Although these scores are noted in the medical chart, they were not analyzed for the purposes of this study. In this study, we assessed measurements in the PACU, at 24h and at seven days]. Patients were discharged with a prescription for oral acetaminophen 325 mg and oxycodone 5 mg and were given detailed instructions for administering pain medications at home every four hours as needed.

Block efficacy, including the need for re-block and inadequate blocks requiring a general anesthetic, was recorded. In addition, any acute complications related to the regional anesthesia technique (e.g., systemic local anesthetic toxicity) were also recorded. Data for patients requiring re-block were included in all analyses and studied separately to examine if re-block contributed to postoperative complications. Data on patients requiring general anesthesia after an inadequate block were also included in this analysis.

### Statistical analysis

All data were stored in an electronic database (FileMaker Pro 3.0v3; Claris Corporation, Santa Clara, CA, U.S.). Descriptive statistics in the form of frequencies, means and standard deviations calculated with 95% confidence intervals were obtained using Intercooled Stata version 8.0 (College Station, TX, U.S.) and Microsoft Excel 2000 (Microsoft Corporation, Seattle, WA, U.S.). Comparisons between TSA and HSA were performed using t-tests for normal continuous variables, Wilcoxon-rank sum tests for nonnormal continuous variables; for categorical variables, Fisher's exact test or Chi-2 analysis.

## RESULTS

Eighty-two patients were enrolled in the 24-month prospective study, most of whom were white (93.4%) and female (62.2%). Table 1 summarizes demographic, anesthesia and surgical characteristics. Mean age was 65.1 ± 12.4 years (interquartile range = 17.6 years). The majority of patients (51.3%) were classified as ASA physical status Class II, 44.9% patients as ASA Class III and 3.9% patients as ASA Class IV. Primary diagnoses, where available, included osteoarthritis (49 patients), humeral fracture (five patients) and avascular necrosis (one patient). Sixty-four patients underwent TSA; HSA procedures were performed in 18 patients. Thirty-three patients underwent a revision surgery. Mean operative surgical time was 214 ± 34 minutes.

A primary regional anesthetic technique was planned and administered in 89.0% (n=73) of patients. Combined regional and general anesthesia was planned and administered in the remaining 11.0% of patients. The following anesthetic techniques were performed: continuous ISB (n=73) and single-injection ISB (n=9). Seven patients (8.5%) in whom an exclusive regional anesthetic approach was planned required general anesthesia intraoperatively. These patients were considered to have failed regional anesthesia. The initial block was considered to be inadequate in six (7.3%) patients, of which five (6.1%) required a re-block and one (1.2%) patient received additional local anesthetic infiltration by the surgeon intraoperatively. Six patients (8.1%) received an intraoperative blood transfusion. Of these, four underwent TSA procedures, one primary HSA procedure and one revision surgery for TSA. No acute complications were reported during the peri-operative period.

**Figure 1:**
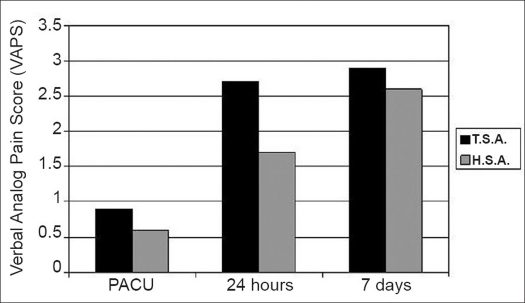
Postprocedural assessment of pain using verbal analog pain score (VAPS). Depicted values represent mean VAPS score at indicated post-procedure time (PACU, 24h, seven days).

**Figure 2:**
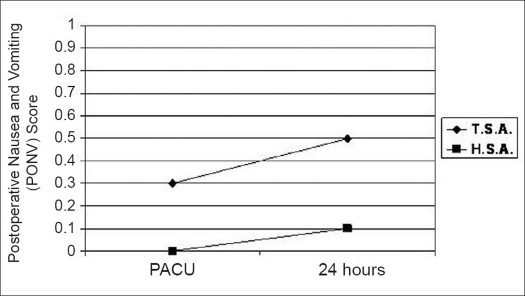
Postprocedural assessment of nausea and vomiting using postoperative nausea and vomiting (PONV) score. Depicted values represent mean PONV score at indicated postprocedure time (PACU, 24h)

The mean PACU VAPS (at rest) was 0.8 ± 2.3 (with movement, 0.9 ± 2.5) and increased at 24h to 2.5 ± 3.1 and at seven days postoperatively to 2.8 ± 2.1 [Figure 1 and Table 2]. Fourteen patients (17.3%) required opioids in the PACU. Of the eight patients reporting pain in the PACU, five patients had required a general anesthetic secondary to an inadequate regional block. The other three patients were considered to have had a successful block for the operative procedure, but complained of pain in the PACU. Patient self-reported nausea and vomiting scores in the PACU were 0.2 ± 1.2 and at 24h 0.4 ± 1.4 [Figure 2 and Table 2], with three patients requiring treatment with an anti-emetic medication. Sixty-one patients received a bolus of local anesthetic via interscalene catheter prior to discharge on postoperative day 1. At the time of discharge, 89% of all patients had an insensate extremity. No postoperative complications were reported due to discharge with an insensate upper extremity. At 24h, 97.0% of respondents (n=66) were extremely satisfied (5 on 1-5 scale) or very satisfied (4 on 1-5 scale) with the anesthesia technique, with mean satisfaction score 4.8 ± 0.6. At seven days, only one patient (n=55 respondents) was dissatisfied with anesthesia, with mean satisfaction 4.8 ± 0.7. At 24h and seven days postoperatively, over 88% of patients in both total and hemi arthroplasty groups indicated that they would choose the same type of anesthesia again.

**Table 2:** Self-reported postoperative pain, nausea, vomiting and satisfaction

	All patients	ISA (n=58)*	HSA(n=17)*	*P*-value
*Outcomes in postanesthesia care unit*				
Verbal analogue pain scale at rest	0 (0,0)	0 (0,0)	0 (0,0)	NS
	0.8 ±2.3	0.8 ±2.5	0.5 ±1.5	
Verbal analogue pain scale with movement	0 (0,0)	0 (0,0)	0 (0,0)	NS
	0.9 ±2.5	0.9 ±2.7	0.6 ±1.7	
Opioids required? (%)				NS
No	82.7	81.0	88.2	
Yes	17.3	19.0	11.8	
Postoperative nausea and vomiting	0 (0,0)	0 (0,0)	0 (0,0)	NS
	0.2 ±1.2	0.3 ±1.4	0±0	
Medications required for postoperative nausea and vomiting? (%)				
No	96.0	96.6	94.1	NS
Yes	4.0	3.4	5.9	
*Outcomes at 24h*				
Verbal analogue pain scale	1 (0,4)	1 (0,4)	0 (0,4)	NS
	2.5 ± 3.1	2.7 ± 3.3	1.7 ±2.4	
Postoperative nausea and vomiting	0 (0,0)	0 (0,0)	0 (0,0)	NS
	0.4 ±1.4	0.5 ±1.5	0.1 ±0.3	
Would you choose the same type of anesthesia? (%)				NS
Yes	97.3	98.3	94.1	
No	2.7	1.7	5.9	
Satisfaction with anesthesia	5 (5,5)	5 (5,5)	5 (5,5)	0.02
	4.8 ±0.6	4.9 ±0.3	4.5 ±1.2	
*Outcomes at seven days*				
Verbal analogue pain scale	3(1,4)	3(1,4)	2.5 (2,4)	NS
	2.8 ±2.1	2.9 ±2.2	2.6 ±1.8	
Would you choose the same type of anesthesia? (%)				NS
Yes	96.0	98.3	88.3	
No	4.0	1.7	11.7	
Satisfaction with anesthesia	5 (5,5)	5 (5,5)	5 (5,5)	NS
	4.8 ±0.7	4.8 ±0.7	4.7 ±0.9	

* Patients who underwent failed regional anesthesia (6 ISA, 1 HSA) were excluded from the analysis. TSA = Total shoulder arthroplasty. HSA = Shoulder hemiarthroplasty. Median (25^th^ percentile, 75^th^ percentile), Mean ± standard deviation, NS = Not significant

In addition, there were no complications in the immediate postoperative period. Eleven patients had to be admitted to the hospital (inpatient setting) postoperatively due to health insurance requirements (i.e., US Medicare requirements mandated that patients undergoing shoulder arthroplasty procedures receive a two-day inpatient stay). At the time of the seven-day follow-up, one patient reported loss of sensation from her shoulder to fingers for the first four days following surgery, after which she reported that her symptoms had resolved completely. No readmissions or other complications resulting from anesthesia were noted at 30 days postoperatively. There were no significant statistical differences in postoperative pain, PONV, satisfaction or complications for patients who had a failed regional anesthetic (required general anesthesia) or who had an inadequate block (required re-block or local anesthetic infiltration by the surgeon).

There were no significant differences in baseline, peri-operative or postoperative characteristics between patients undergoing TSA and HSA procedures. Patients undergoing HSA were noted to have a lower surgical time (192 ± 34 vs. 220 ± 31 min; *P*<0.01) compared to the TSA group. In addition, patients undergoing TSA were noted to have a higher satisfaction with anesthesia at seven days postoperatively (4.9 ± 0.3 vs. 4.5 ± 1.2; *P*=0.02). No other significant differences were noted in self-reported postoperative pain scores, PONV scores and satisfaction in the PACU, at 24h and seven days.

## DISCUSSION

The results of this study suggest that regional anesthesia for ambulatory shoulder arthroplasty provides adequate analgesia and high patient satisfaction. In comparing patients undergoing TSA versus HSA, there were no major differences in outcomes with respect to postoperative pain, PONV or patient satisfaction scores. Despite concerns about discharging patients with an anesthetized upper extremity, neither shoulder trauma nor dislocation secondary to an insensate upper extremity was seen postoperatively in our patient sample.

The incidence of PONV in this study was low in the PACU and at 24h postoperatively for both the total and hemi arthroplasty groups. As no patients received prophylaxisantiemetic medication, these self-reported scores were remarkably low. These results are in accordance with a recent study by Hadzic *et al.*, which reported similarly low PONV scores.^7^ In addition, no patients were readmitted to the hospital after discharge for complications related to anesthesia. Our results were consistent with previous reports^6^,^7^ that unplanned readmissions due to complications such as nausea and vomiting were lower after orthopedic procedures performed under regional anesthesia compared to general anesthesia. This was particularly encouraging when compared to the high incidence of such complication following ambulatory surgery.^20^–^22^

Despite the lack of a control group with patients receiving general anesthesia, the low pain scores observed in the postoperative period suggest that ambulatory shoulder arthroplasty can be successfully performed with the use of regional anesthesia. Several studies have reported improved pain with regional anesthesia compared to general anesthesia.^7^,^8^ Previous studies have documented analgesia lasting 12-14 postoperative hours with a single-injection peripheral nerve blockade.^23^,^24^ In this series of patients, continuous regional anesthesia was used to extend the neural blockade in the ambulatory surgery setting, including the 23h observation unit. Recent reports suggest a significant need (200/212 patients, 92%) of parenteral opioids for pain control following shoulder procedures performed under ISB;^25^ our experience suggests excellent pain control with only 17.3% requiring opioids in the PACU.

Although shoulder procedures are susceptible to neural injury,^26^,^27^ possibly due to partially or completely insensate extremity secondary to brachial plexus blockade, these complications are uncommon and usually resolve within months.^28^ Most patients in our cohort (89%) were discharged with an insensate extremity; however, there was only one reported patient complication of residual paresthesia for several days after surgery in the extremity that underwent ISB. In addition, all patients in our series were classified as ASA physical status Class II or greater with a mean age of 65.1 years. This higher anesthetic risk is typical for older patients requiring major surgical procedures. Despite the higher anesthetic and surgical risks associated with greater co-morbidities, our study demonstrates a low rate of postoperative complications, which differs from a recent report by Weber and Jain.^25^ Our results are in accordance with several previous reports, which have demonstrated a reduction in the risk of complications for selected procedures performed in elderly patients under regional anesthesia.^29^–^31^ This low incidence of complications coupled with the patient's own interest in being discharged home early is reflected in our overall high rate of patient satisfaction when evaluated postoperatively at 24h and seven days. Patient satisfaction in this sample is consistent with previous reports of high patient satisfaction with regional anesthesia for shoulder surgery.^7^,^9^–^11^ Accordingly, no patients in our study had to be admitted to the hospital for complications related to surgery or anesthesia. Eleven patients were admitted as inpatients immediately following surgery to comply with US Medicare requirements; however, no complications were noted during the 30-days postoperative period. Based on the high level of analgesia and patient satisfaction of subjects in this study, an early, safe discharge with the use of regional anesthesia should be considered in patients undergoing shoulder arthroplasty procedures in the ambulatory setting.

As this was a consecutive series of patients, anesthetic technique was left to the discretion of the attending anesthesiologist, with the patient receiving either a continuous or single-injection ISB, combined (11%) or not (89%) with general anesthesia. As such, our small cohort of 82 patients warrants further study in a larger sample of patients. A primary limitation of this study is the lack of a control group of receiving only general anesthesia. Such a group was not deemed feasible, given the high demand for ambulatory procedures and regional anesthesia at our institution. Based on our results, further evaluation of patient postoperative pain, PONV and satisfaction in a large cohort of patients randomized to undergo either regional or general anesthesia is warranted. In addition, due to the relatively small number of patients in our study, we were unable to evaluate outcomes of a planned combined regional and general anesthesia approach, which has been demonstrated to be superior to general anesthesia alone.^32^ With regards to data collection, we were limited to assessing pain in the PACU, at 24h and at seven days in our electronic ambulatory anesthesia database; it is quite possible that we were unable to assess fluctuating pain levels in the interim time between these three assessments. Lastly, as the data was not entirely colleted by the physician researchers themselves, the possibility of assessment bias exists in our analysis.

In essence, our findings demonstrate that regional anesthesia for shoulder arthroplasty procedures performed in the ambulatory setting is promising, with a low occurrence of postoperative pain, PONV and complications, all while achieving high patient satisfaction.
